# Atorvastatin-Induced Autoimmune Hepatitis: A Case Report

**DOI:** 10.7759/cureus.47807

**Published:** 2023-10-27

**Authors:** Jonathan Tse, Sam Natla, Rohit Mekala, Ian Crumm, Melissa H Olken

**Affiliations:** 1 Internal Medicine, Western Michigan University Homer Stryker M.D. School of Medicine, Kalamazoo, USA; 2 Internal Medicine/Pediatrics, Western Michigan University Homer Stryker M.D. School of Medicine, Kalamazoo, USA

**Keywords:** statin side effects, statin-induced autoimmune hepatitis, statin safety, idiopathic autoimmune hepatitis, drug-induced liver injury, drug-induced autoimmune hepatitis, atorvastatin-induced autoimmune hepatitis

## Abstract

Drug-induced autoimmune hepatitis (DIAIH) is a poorly understood form of drug-induced liver injury that presents with features mimicking autoimmune hepatitis. Statins, commonly prescribed for lowering cholesterol and for cardiovascular disease prevention, have been documented in rare cases as being responsible for DIAIH. In this case report, we detail a case where a patient developed DIAIH due to her atorvastatin. We also highlight the diagnostic approach and management strategies for DIAIH.

## Introduction

While drug-induced liver injury (DILI) is relatively well documented, a smaller subset of these patients may present with drug-induced autoimmune hepatitis (DIAIH), a poorly understood DILI with features mimicking idiopathic autoimmune hepatitis (AIH) [1-4]. Nitrofurantoin and minocycline have been most commonly associated with DIAIH but other medications like statins have also been documented in rare cases as being responsible for DIAIH [1-4]. Statins are 3-hydroxy-3-methylglutaryl coenzyme A (HMG-CoA) reductase inhibitors commonly prescribed as a cholesterol-lowering agent and for cardiovascular disease prevention that are generally known for their favorable side effect profile [5]. We report a case where a patient developed DIAIH while taking atorvastatin.

## Case presentation

The patient was a 74-year-old female with iron deficiency anemia, peripheral arterial disease, and hyperlipidemia presenting with a one-month history of jaundice and bilateral lower extremity edema. Further review of symptoms was negative. The patient denied any alcohol, tobacco, recreational drug, or supplement use. All other family and social histories were noncontributory. Her home medications included ferrous sulfate, atorvastatin, lisinopril, and clopidogrel.

Two years prior, she had significant elevations in liver function tests (LFTs): aspartate aminotransferase (AST) 199 U/L, alanine aminotransferase (ALT) 385 U/L, alkaline phosphatase (ALP) 163 U/L, and total bilirubin (TBili) within normal limits (WNL). Her previous baseline hepatic function panels had always been normal. During the episode two years earlier, further diagnostic testing was negative, including a liver ultrasound, antimitochondrial antibody (AMA), anti-smooth, alpha-1 antitrypsin, ceruloplasmin, and ferritin. Antinuclear antibody (ANA) was 1:640, but Rheumatology felt that it was insignificant. The episode ultimately resolved three months after her atorvastatin was temporarily held.

Following the resolution of this first episode of elevated LFTs, the patient restarted her atorvastatin and took it for a total of 20 months. Two months prior to admission for her second episode of elevated LFTs, the patient’s primary care physician noticed mild elevations in LFTs (AST 75 U/L, ALT 95 U/L, ALP WNL, and TBili WNL), so atorvastatin was once again held. Serum protein electrophoresis demonstrated polyclonal gammopathy with decreases in albumin (2.7 g/dL) and alpha-2 globulin (0.4 g/dL) and an increase in gamma globulin (3.0 g/dL). Repeat liver ultrasound showed normal liver with mild ascites. Due to her uptrending LFTs (AST 262 U/L, ALT 127 U/L, ALP 345 U/L, TBili 21.9, and direct bilirubin (DBili) 15.6 mg/dL), her gastroenterology (GI) provider recommended admission for inpatient workup.

**Figure 1:**
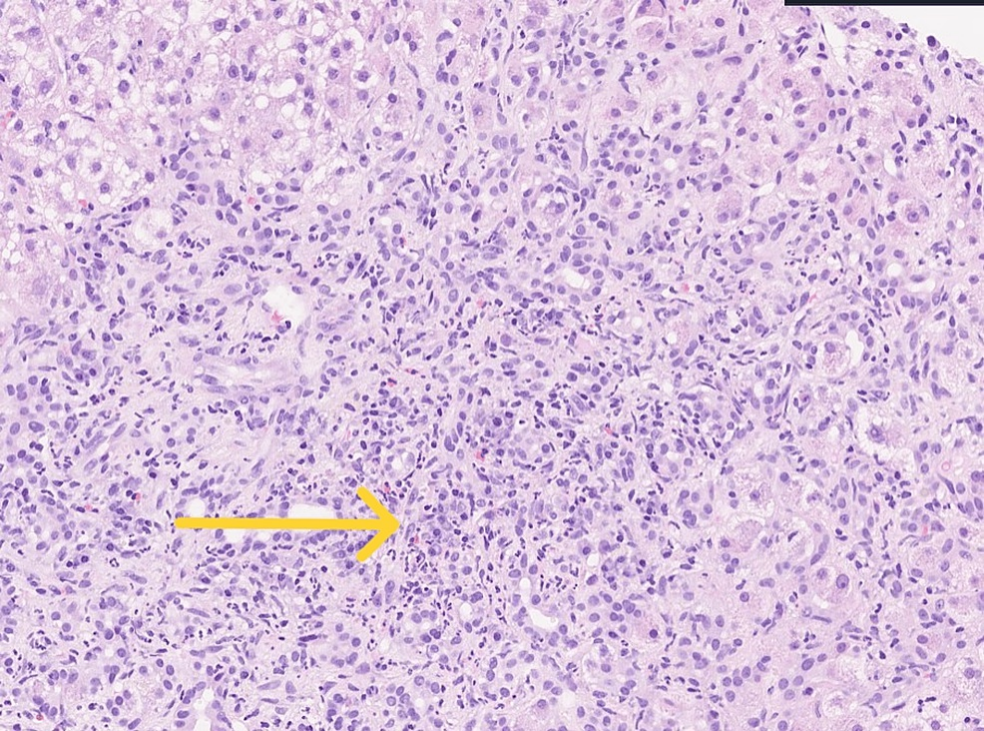
Mixed periportal necroinflammatory infiltrate with increased plasma cells and ductular reaction compatible with autoimmune drug-induced liver injury

On admission, three days later, vitals were within normal limits. Physical examination was significant for scleral icterus, diffuse jaundice, and an unremarkable abdomen exam. Laboratory findings were significant for hypokalemia (2.7 mmol/L), elevated ALP (346 U/L), AST (207 U/L), ALT (122 U/L), DBili (16.2 mg/dL), TBili (22.7 mg/dL), prothrombin time (PT)/international normalized ratio (INR) (25.1/2.1), and D-dimer (3.61 ug/mL), and decreased fibrinogen (98 mg/dL). This resulted in a Child-Pugh Class C and Model for End-Stage Liver Disease (MELD) score of 26. The anti-liver-kidney microsomal type 1 (Anti-LKM-1) antibody was negative. The autoimmune panel was significant for ANA of 1:160 and positive ribonucleoprotein (RNP) antibody. While she was hepatitis C antibody positive, a hepatitis C polymerase chain reaction (PCR) test was negative, indicating either a false positive or a prior infection. Magnetic resonance cholangiopancreatography (MRCP) showed cirrhosis without masses or evidence of biliary obstruction, with potential functional obstruction due to a twisting cystic duct. During her hospital course, Interventional Radiology performed a random liver biopsy. She was discharged after four days in stable condition and with downtrending LFTs (AST 139 U/L, ALT 82 U/L, ALP 307 U/L, and TBili 20.6 mg/dL) while still holding her atorvastatin. Pathology showed chronic hepatitis with periportal fibrosis and lymphoplasmacytic infiltrate with bile ductular proliferation (Figure 1).

## Discussion

Our patient presented at admission with nonspecific findings for liver disease like scleral icterus and jaundice. With impressive elevations in bilirubin, aminotransferases, and ALP, the patient presented with a mixed picture of cholestatic and hepatocellular injury. A thorough workup was conducted, ruling out other common and possible causes of hepatic dysfunction like viral hepatitis, alcoholic hepatitis, nonalcoholic fatty liver disease, Wilson’s disease, hemochromatosis, primary sclerosing cholangitis, primary biliary cholangitis, and biliary obstruction.

As previously mentioned, DIAIH can present with many features that mimic AIH [1]. Our patient’s autoimmune panel was positive for ANA of 1:160 and her serum protein electrophoresis demonstrated hypergammaglobulinemia. During the patient’s admission, an Interventional Radiology-guided liver biopsy was performed which demonstrated many autoimmune-like features on histology including interface hepatitis, lymphoplasmacytic infiltrates, numerous plasma cells, severe portal inflammation, and focal periportal fibrosis (Figure 1) [6,7]. Based on these findings, the Simplified Autoimmune Hepatitis Score was 8, which would be considered definitively diagnostic of autoimmune hepatitis (Table 1). During the present episode of liver injury, the patient’s atorvastatin was withdrawn, but she did not receive corticosteroid therapy. Approximately two months after statin cessation, the patient's LFTs downtrended. AIH requires corticosteroid or immunosuppressive therapy for resolution and remission, whereas DIAIH may occasionally resolve merely with the withdrawal of the offending agent [2-4,8]. The use of corticosteroids in patients with DIAIH is often made on a per-patient basis and there are currently no set management guidelines for corticosteroid therapy in DIAIH [3,4,9]. Furthermore, DIAIH is often also differentiated from AIH by sustained remission of symptoms following discontinuation of corticosteroid therapy after resolution of liver dysfunction [2,4]. Given the patient’s jaundice and the aminotransferase levels being greater than five times the upper limit of normal, according to the American College of Gastroenterology guidelines, permanent cessation of her atorvastatin is strongly recommended [9]. Unfortunately, the patient passed away after discharge due to unrelated causes, leaving it unknown if drug cessation alone would have led to sustained remission. However, improvement of symptoms with statin cessation alone makes DIAIH the more likely diagnosis. More conclusively, the Roussel Uclaf Causality Assessment Method (RUCAM) can be used to assess the likelihood of atorvastatin as the causative agent. Of note, the RUCAM score is limited by a subjective scoring system and complicated instructions; but it remains the most widely accepted method of assessing causality in DILI [10]. The patient had a RUCAM score of 6, indicating that atorvastatin would be considered the probable causative agent of her liver injury, leading to our diagnosis of atorvastatin-induced AIH (Table 2).

**Table 1:** Simplified Autoimmune Hepatitis Score Breakdown ANA/SMA: Antinuclear antibodies/smooth muscle antibodies; LKM1 antibody: Liver kidney microsomal type 1 antibody

Parameters	Points
ANA/SMA	+2
LKM1 Antibody	0
IgG	+2
Liver Histology	+2
Viral Hepatitis Absent	+2
Total	8

**Table 2:** Roussel Uclaf Causality Assessment Method (RUCAM) Score Breakdown Current Admission: R value=3.32; Prior Episode of Elevated LFTs: R value=7.47; R value: (ALT ÷ ULN ALT)/(ALP ÷ ULN ALP) LFTs: Liver function tests; ALT/ALP: Alkaline phosphatase/alanine aminotransferase; ULN: Upper limit normal

Parameters	Current Admission	Prior Episode of Elevated LFTs
Time to Onset	+1	+1
Change in ALT/ALP Between Peak Value and ULN	+1	+2
Risk Factors (Alcohol/Pregnancy, Age)	+1	+1
Concomitant Drugs	0	0
Exclusions of Other Causes of Liver Injury	+1	+1
Previous Information on the Hepatotoxicity of the Drug	+2	+2
Response to Readministration	0	+3
Total	6	10

Two years prior also, the patient had an unexplained episode of elevated liver enzymes and a positive ANA of 1:640. Serum electrophoresis and liver biopsy were not conducted at that time. Otherwise, a thorough workup was done with no etiology identified. The episode resolved within three months after the atorvastatin was held. The relatively quick resolution within months is consistent with other cases of DIAIH in the literature [4]. Moreover, given the complete resolution of symptoms with drug cessation alone, the likely etiology was DIAIH. It is unknown how long the patient had been taking her atorvastatin prior to this episode but she restarted it for 20 months between these two episodes of DIAIH. The latency to onset of DIAIH following exposure is highly variable: ranging from weeks to years after drug administration [2-4]. The recurrence of DIAIH following the reintroduction of atorvastatin further supports the diagnosis of atorvastatin-induced AIH [3,4]. A RUCAM score of 10 indicates that atorvastatin was the highly probable causative agent during this episode of DIAIH (Table 1).

## Conclusions

Overall, it is important to remember that statins are both safe and efficacious. After all, statin-induced autoimmune hepatitis is exceedingly rare. However, this case emphasizes the importance of regular liver function evaluation and follow-up following an episode of unexplained elevated liver enzymes. More importantly, it underscores the importance of holding a statin in any case of suspected liver injury.
